# Supplementary material for: SARS-CoV-2 escape from cytotoxic T cells during long-term COVID-19
Source: Nat Commun. 2023 Jan 10;14:149. doi: 10.1038/s41467-022-34033-x (PMC9831376; doi:10.1038/s41467-022-34033-x)
Supplement: Supplementary file 1 — Supplementary Information [file 41467_2022_34033_MOESM1_ESM.pdf]

Supplementary Information

Supplementary Note 1

Patient's BMI was 18.07, blood group - 0 (I), Rh+. Concomitant diagnoses did not include diabetes, cardiovascular diseases, anamnesis of stroke, acute myocardial infarction, thromboembolism, or chronic lung obstruction disease. Between June and September, 2020, there could be an invasive mycoses of lungs, but mycological assessment did not reveal any mycotic agents except *Candida* spp. There was no candidemia or bacteremia. Vancomycin-resistant Enterococcus (VCE) was detected in stool specimens during hospitalization in June-August 2020 and January 2021 but did not cause bacteremia. After bone marrow transplantation, no infection was detected by PCR, bacteriological or mycological expertise, or specimen’s microscopy for infectious agents, besides VCE in stool. Patient S did not leave the Russian Federation between the beginning of 2020 and the end of SARS-CoV-2 infection, and had no pets at home during that period.

Supplementary Table 1: The timeline of patient S survey and therapy.

Event or procedure	Dates and results	Comment
Contact with patient A	April 10, 2020 - April 16, 2020	Shared a ward in a hospital
SARS-CoV-2 PCR tests	In 2020: April 17 (+), April 30 (+), May 14 (-), May 19 (-), June 9 (-), July 14 (-), August 3 (+), August 5 (+), August 8 (+), August 11 (+), August 13 (+), August 17 (+, Ct 22), August 20 (+, Ct 19), August 21 (+), August 26 (+), August 27 (+), August 31 (+), September 2 (+, Ct 32), September 3 (+), September 8 (+), September 12 (-), September 16 (-), November 10 (-), December 16 (-);<br>In 2021: January 9 (+), January 11 (+), January 15 (+), January 19 (+, Ct = 21), January 22 (+, Ct = 24), January 22 (+, Ct = 31), February 1 (+, Ct = 24), February 8 (+, Ct = 30), February 16 (+, Ct = 19), February 19 (+, Ct = 29), March 1 (+, Ct = 32), March 10 (-), April 5 (-)	(+) - positive PCR;<br>(-) - negative PCR;<br>Ct - real time PCR cycle threshold when known..
Periods of pneumonia	In 2020: June 6 - September 1;<br>In 2021: January 9 - February 1.	
ELISA for anti-S-SARS-CoV-2 IgG	In 2020:<br>August 17, 2020: negative (Cut-off-Index 0.54);<br>November 12, 2020: positive (Cut-off-Index 3.75);<br>December 15: ambiguous (Cut-off-Index 1.03).	Cut-off-index for ELISA: <0,8 – negative; 0,8 – 1,1 – ambiguous; >1 - positive.
VN assay	In 2020:<br>August 17: neutralizing antibodies not detected (titer <10);<br>November 12: neutralizing antibodies not detected (titer <10);<br>December 15: neutralizing antibodies not detected (titer <10).	
Sequenced samples	Patient A: April 19, 2020;<br>Patient S: In 2020: August 17, August 20;<br>In 2021: January 11, January 19, January 22, February 19.	The January 11, 2021 swab was obtained prior to convalescent plasma transfusion on the same date.
Blood samples	August 20, 2020; February 16, 2021	
Chemotherapy	In 2020:<br>CHOP: January 5 - January 9; R-EPOCH: January 24 - January 29; R-EPOCH: February 13 - February 18; R-EPOCH: March 5 - March 10; R-ICE: April 5 - April 8; R-ICE: June 23 - June 27; R-ICE: July 24 - July 28; R-GemOx: October 30; GemOx: October 30; GemOx: November 14; ICE: December 5 - December 12; BEAM: December 21 - December 27.	Abbreviations of chemotherapy regimens are standardized for DBCL[1].
Autological transplantation of hematopoietic stem cells (Auto-HSCT)	December 28, 2020	
Convalescent plasma	In 2021: January 11, January 15, January 18.	

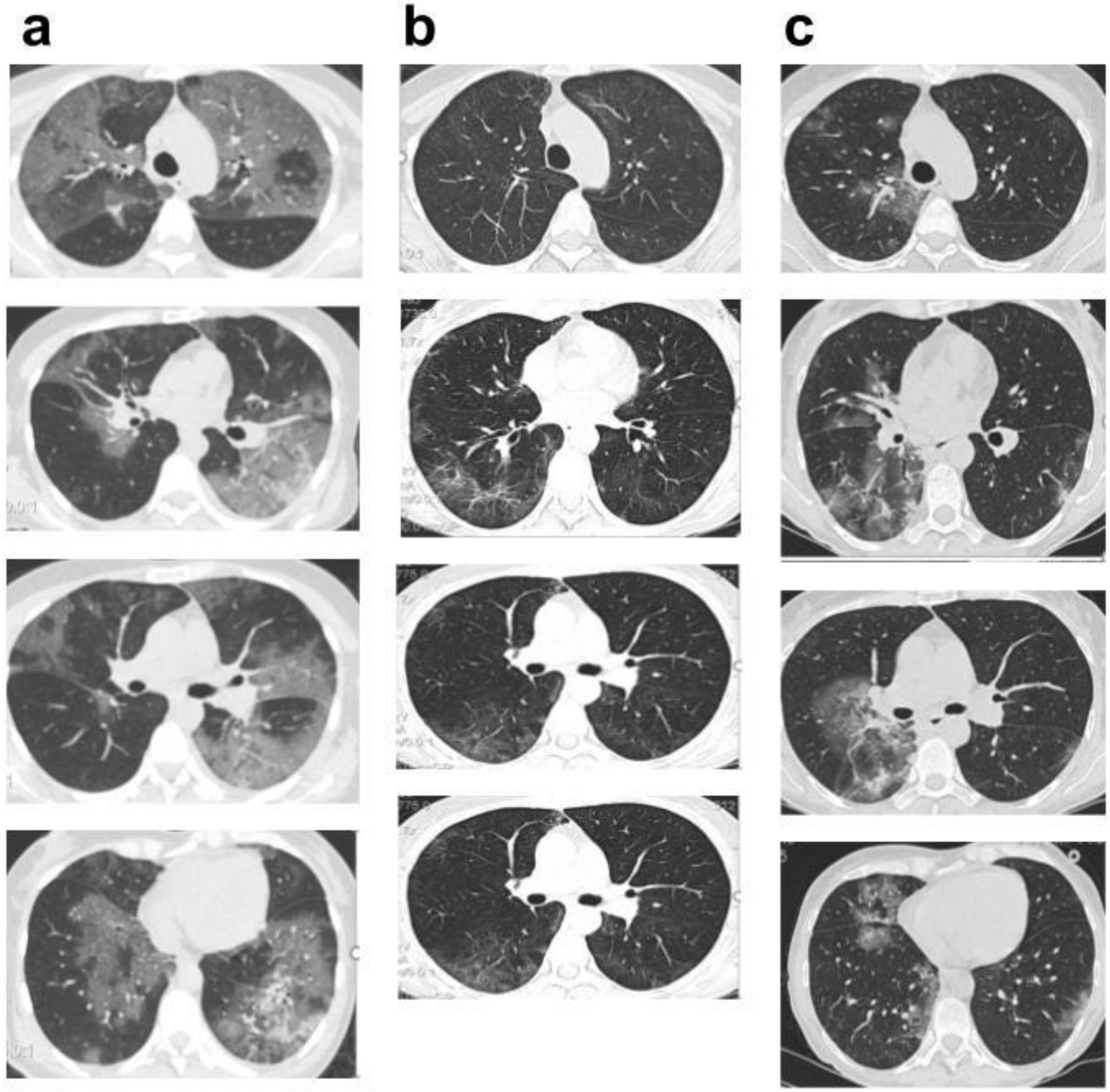

Supplementary Fig. 1 CT scans of patient S lungs obtained by Optima 660 (GE) CT-scanner with standard in-house protocols without contrast enhancement in June 2020 (**a**), August 2020 (**b**) and January 2021 (**c**). In both lungs in **(a), (b), (c)** there were bilateral, multifocal and diffuse ground-glass opacifications with small regions of subpleural consolidations, but without predominant distribution in **(a)**, mild reticulation and regions of architectural distortion with the formation of subpleural bands in **(b)**, small regions of mild reticulation, vascular dilatation, and regions of linear consolidation with the formation of bands in **(c)**. CT-patterns can be determined as typical for COVID-19 pulmonary disease according to the Radiological Society of North America expert consensus [2].

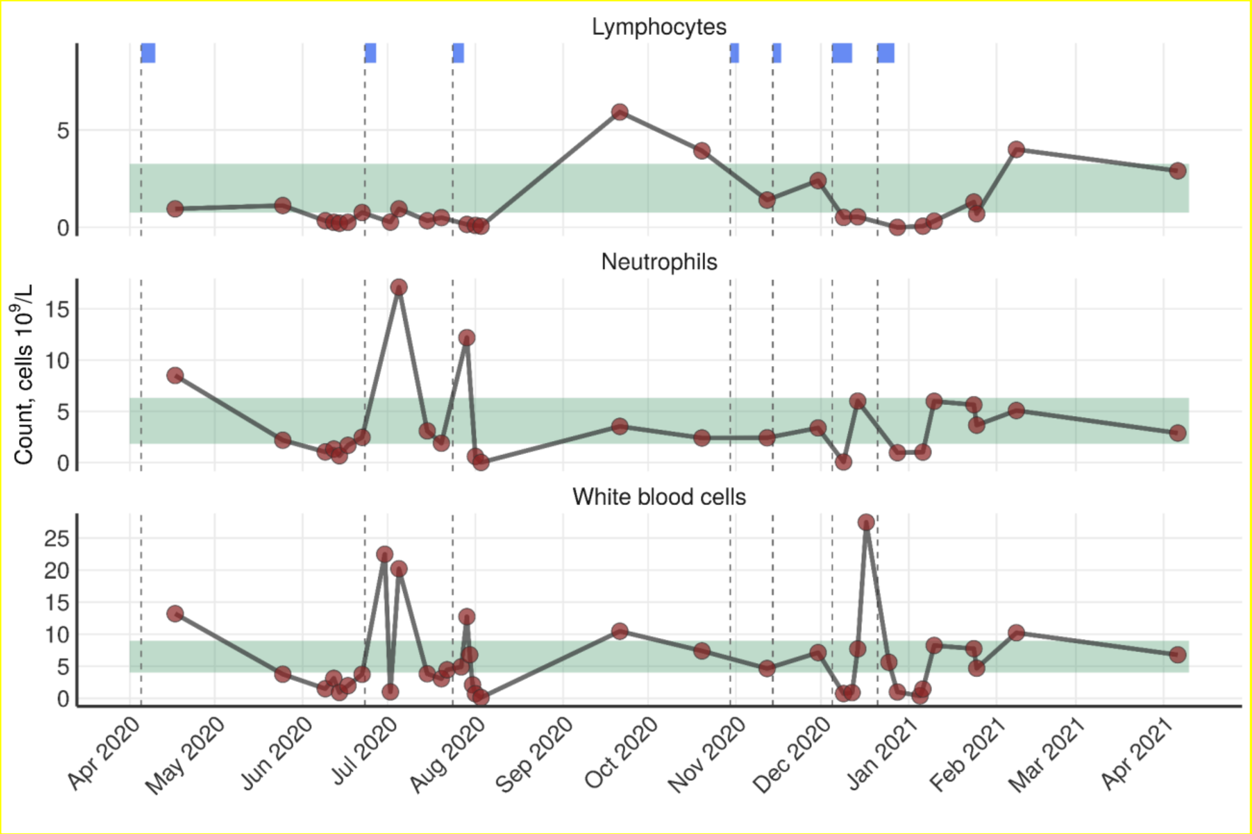

Supplementary Fig. 2. Cell counts of lymphocytes, neutrophils and white blood cells in 10^9/L observed in patient S between April 17, 2020 and June 4, 2021. Green shading indicates the normal range for females of the corresponding age in each category respectively. Blue bars and dashed lines indicate courses of chemotherapy; chemotherapy regimens are described in Supplementary Table 1 and Fig. 1a. Source data for are provided as a Source Data file.

Supplementary Note 2

Between 19 and 47 genetic changes distinguish the patient S samples from the Wuhan-Hu-1/2019 reference strain [3]. Seven of these changes, including the three SNPs at adjacent positions 21881-21883, were contained in each of the patient S samples, placing them in the B.1.1 lineage. The lineage of patient S carries the remaining 12 to 40 genetic changes. The patient A sample carries the seven mutations characteristic of B.1.1 but no other mutations, confirming patient A as the likely source of infection for patient S, and indicating that the remaining changes are specific to patient S.

Supplementary Note 3

Among the 12 mutations specific to patient S and observed in both August 2020 samples, 10 were single-nucleotide mutations (6 nonsynonymous, 3 synonymous and 1 creating a premature stop codon), and the remaining 2 were in-frame deletions. In the second August 2020 sample, 6 additional changes (4 nonsynonymous, 1 synonymous and 1 in-frame deletion) reached consensus frequencies.

Six of the mutations that reached consensus frequencies in the August 2020 samples reversed back to the ancestral state by January 2021, including the ΔF combination (see Supplementary Note 4). Additionally, the January-February 2021 samples gained 21 new mutations compared to the August 17, 2020 sample. 10 of these mutations (6 nonsynonymous and 4 synonymous) were detected in all winter samples. The other 11 mutations (8 nonsynonymous and 3 synonymous) were each called in a subset of the winter samples; in the remaining samples, the corresponding sites were usually poorly covered. Overall, 34 changes were observed in the January 22, 2021 sample, which is the highest-quality sample among the winter 2021 samples (Fig. 1d, Supplementary Table 2). Together with the six reverted changes, this totals to 40 observed changes.

In addition to changes in the consensus sequence, we observed a number of variants at intermediate frequencies (above 30% in at least one of the samples, but below 50% in all samples and therefore not included in the consensus sequence; Fig. 1d, Supplementary Table 2), indicating within-host polymorphism. Three such variants (1 nonsynonymous and 2 synonymous) were observed in the August samples (all of them were lost in the January-February samples), and three (2 synonymous and 1 frame-disrupting deletion) were observed in the January-February samples (all absent in the August samples).

Supplementary Table 2: **The list of mutations and their frequencies in sequencing reads obtained from patient S swab samples.** Only variants reaching 30% frequency at least in one of the samples are shown. The consensus variants (read frequency > 50%) are highlighted in blue, nonsynonymous nucleotide substitutions are in bold. NC (no coverage) indicates coverage depth less than 4 reads. “Selection” and “Trend Z” columns mark positions that experience positive selection and increase of corresponding changes in frequency, according to observablehq.com[4] accessed on 31th March 2020. Source data for are provided as a Source Data file.

Gene	Nucleotide change	AA change	Aug 17 2020	Aug 20 2020	Jan 11 2021	Jan 19 2021	Jan 22 2021	Feb 19 2021	Selection	TrendZ
leader	C:676:T	leader:G137G	0.000	0.000	0.992	0.990	0.998	1.000		
	G:1312:A	nsp2:L169L	0.000	0.000	0.998	0.998	0.995	0.999		
	C:1441:T	nsp2:G212G	0.995	0.995	0.996	0.996	0.995	0.992		
	T:1552:C	nsp2:A249A	0.000	0.000	0.999	0.996	0.998	0.996		
nsp2	**C:2037:T**	**nsp2:A411V**	**0.000**	**0.000**	**0.996**	**0.996**	**0.999**	**1.000**	+	
	**A:4229:G**	**nsp3:T504A**	**0.434**	**0.730**	**0.000**	**0.000**	**0.000**	**0.000**		
	**A:4229:C**	**nsp3:T504P**	**0.000**	**0.000**	**0.994**	**1.000**	**0.999**	**1.000**		
	**G:5180:A**	**nsp3:D821N**	**1.000**	**1.000**	**1.000**	**1.000**	**1.000**	**NC**		
nsp3	**C:7086:T**	**nsp3:T1456I**	**0.000**	**0.000**	**1.000**	**1.000**	**1.000**	**NC**	+	+
	T:9091:C	nsp4:S179S	0.000	0.000	0.000	0.000	0.000	0.333		
	**C:9438:T**	**nsp4:T295I**	**0.995**	**1.000**	**0.997**	**1.000**	**0.999**	**1.000**		
nsp4	**G:9497:A**	**nsp4:V315I**	**0.974**	**1.000**	**0.937**	**0.958**	**0.916**	**NC**		+
3C	G:10318:A	3C:K88K	0.014	0.460	0.000	0.000	0.000	0.000		
	TG:11082:T	nsp6:del37	0.000	0.005	0.302	0.221	0.238	0.175		
	**G:11083:T**	**nsp6:L37F**	**0.006**	**0.007**	**0.621**	**0.743**	**0.685**	**0.810**		
nsp6	**G:11804:A**	**nsp6:V278I**	**0.000**	**0.000**	**0.000**	**0.000**	**0.000**	**0.480**		
nsp7	**T:12015:G**	**nsp7:V58G**	**0.997**	**0.998**	**1.000**	**0.999**	**0.999**	**0.999**		
nsp9	A:12886:G	nsp9:T67T	1.000	0.993	0.997	0.999	0.998	1.000		
	C:13620:T	RdRp:D60D	0.453	0.222	0.000	0.000	0.000	NC		
	**A:13913:G**	**RdRp:N158S**	**0.000**	**0.000**	**1.000**	**0.998**	**0.999**	**NC**		
	C:14625:T	RdRp:C395C	0.000	0.000	0.000	0.000	0.000	0.391		
RdRp	A:15456:G	RdRp:S672S	0.000	0.000	1.000	0.997	1.000	1.000		
	C:16575:T	helicase:D113D	0.000	0.000	1.000	1.000	1.000	NC		
helicase	A:17337:G	helicase:T367T	0.000	0.000	1.000	0.999	0.996	NC		
endornase	**C:20234:T**	**endornase:P205L**	**0.000**	**0.000**	**1.000**	**1.000**	**1.000**	**1.000**	+	+
	**C:21711:T**	**S:S50L**	**1.000**	**0.998**	**1.000**	**1.000**	**1.000**	**1.000**		
	ATACATG:21764:A	S:del68_70	0.354	0.626	0.000	0.000	0.000	0.000	+	+
	TTTTGGGTGTTTA:21981:T	S:del140_144	1.000	0.922	0.962	0.965	0.967	1.000	+	+
	**G:22381:T**	**S:R273S**	**NC**	**0.000**	**1.000**	**NC**	**1.000**	**NC**		
	C:22675:A	S:S371S	NC	0.636	0.000	NC	0.000	NC		
	**T:22882:G**	**S:N440K**	**NC**	**0.000**	**NC**	**NC**	**1.000**	**NC**	+	+
	**T:22917:G**	**S:L452R**	**NC**	**0.000**	**NC**	**NC**	**1.000**	**NC**		+
	**A:22920:T**	**S:Y453F**	**NC**	**0.625**	**NC**	**NC**	**0.000**	**NC**		
	**C:22971:A**	**S:T470N**	**NC**	**0.992**	**NC**	**1.000**	**1.000**	**NC**		
	**G:22988:A**	**S:G476S**	**NC**	**0.000**	**NC**	**1.000**	**1.000**	**NC**	+	
	**C:23423:T**	**S:P621S**	**0.349**	**0.322**	**0.000**	**0.000**	**0.000**	**0.000**		
S	**A:23772:G**	**S:D737G**	**0.999**	**1.000**	**0.991**	**0.999**	**1.000**	**0.989**		

ORF3a	T:25435:C	ORF3a:L15L	0.000	0.000	0.000	0.530	0.239	1.000		
E	**C:26261:T**<br>**T:26320:C**<br>T:26445:C	**E:S6L**<br>**E:F26L**<br>E:S67S	**0.000**<br>**0.000**<br>1.000	**0.060**<br>**0.000**<br>1.000	**1.000**<br>**NC**<br>NC	**1.000**<br>**1.000**<br>1.000	**1.000**<br>**1.000**<br>1.000	**1.000**<br>**NC**<br>NC		
M	**T:26908:G**	**M:L129R**	**0.186**	**0.047**	**1.000**	**1.000**	**0.967**	**NC**		
ORF6	T:27351:C	ORF6:S50S	0.000	0.000	0.968	1.000	1.000	NC		
ORF7a	GATT:27758:G	ORF7a:del2	1.000	1.000	1.000	1.000	1.000	NC		
ORF8	**C:27945:T**	**ORF8:Q18***	**1.000**	**0.995**	**1.000**	**0.987**	**1.000**	**NC**		
N	**C:28289:A**<br>**A:28856:G**	**N:P6T**<br>**N:R195G**	**0.140**<br>**0.428**	**0.061**<br>**0.702**	**0.993**<br>**0.000**	**0.993**<br>**0.000**	**0.997**<br>**0.000**		**+**	

Supplementary Note 4

Among the positions that acquired amino acid mutations, ten (nsp2:A411V, nsp3:T1456I, nsp4:V315I, endornase:P205L, S:del68_70, S:del140_144,S:N440K, S:L452R, S:G476S, N:R195G) experienced pervasive positive selection according to the FEL (fixed effects likelihood) model[5] and/or their frequencies grew in the global viral population according to Jonckheere's trend test (Supplementary Table 2), as reported in ref.[4] (accessed on 31th March 2020).

Many of the detected mutations are known from other studies. Notably, these include the ΔF combination (S:Y453F + S:Δ69-70HV), which is observed in the consensus of the August 20, 2020 sample; S:Y453F is also found at high read frequency in the other 2020 sample, indicating that ΔF was probably also present at this time point (S:69-70 is too poorly covered at this time point to be called). The ΔF combination was previously described as associated with mink-related clusters. It has arisen in parallel in multiple mink populations; among humans, it was mainly found in cases traceable to minks ("Cluster 5", or B.1.1.298), indicating reverse transmission [6]. Despite the presence of the ΔF combination, patient S cannot be placed into the cluster 5 clade because cluster 5 is separated from B.1.1 by two additional mutations (those at positions 15656 and 25936) which are absent in patient S (Supplementary Fig. 3). Furthemore, the ΔF combination was not fixed in the August 2020 samples of patient S but segregated at an intermediate frequency (Supplementary Table 2). Together, this indicates that the ΔF combination was acquired by patient S independently. It was not observed in any of the 2021 patient S samples, indicating that it had been reversed by that time.

The ΔF combination confers the ability to rapidly replicate to high titers and to evade recognition by neutralizing antibodies[7], raising concerns that these mutations may affect vaccine efficiency. Y453F affects the receptor-binding domain (RBD), possibly increasing hACE2 binding[8,9]. It allows immune escape from monoclonal antibodies and polyclonal sera; in particular, it has led to 57% escape from the REGN10933 monoclonal antibody, a component of FDA-approved Regeneron's REGN-COV2 cocktail for treatment of COVID-19 patients, although it did not

allow escape from the full cocktail of two antibodies (REGN10933+REGN10987)[10]. It was also shown to escape cellular immunity in HLA-A24-restricted patients[11].

The second mutation of the ΔF combination, S:Δ69-70HV, was recently shown to occur in a virus from another immunocompromised patient with COVID-19[12] (Supplementary Fig. 7). In that study, S:Δ69-70HV has been fixed during convalescent plasma therapy, suggesting antibody selection pressure, which is consistent with decreased virus sensitivity to neutralization with sera from recovered patients. However, patient S was not treated with convalescent plasma in 2020 and had no detectable neutralizing antibody response, suggesting that S:Δ69-70HV could have been favored by some other factor of selection. In patient S, both the S:Y453F and the S:Δ69-70HV mutations were polymorphic in 2020 and were lost by 2021, suggesting that this other factor may have been transient. The presence of the ΔF combination in the August 19, 2020 sample may underlie reduced sensitivity to neutralizing antibodies for this time point (Supplementary Fig. 4). Reacquired sensitivity to neutralizing antibodies by February 19, 2021 is also consistent with the loss of the ΔF combination by this time (Supplementary Fig. 4).

Besides S:Δ69-70HV, patient S has acquired six additional mutations that were also observed in other immunocompromised patients: S:S50L[13], S:N440K[14], S:Δ141-144[13–17], nsp3:T504I[15], nsp3:T295I[13] and nsp6:L37F[14] (Supplementary Fig. 7). The most recurrent of these mutations, S:Δ141-144, was shown to lead to an escape from neutralizing antibodies[18]. It falls into the recurrent deletion region[18] where frequent deletions are observed, including S:Y144del, the lineage defining deletion of B.1.1.7[19] which is speculated to have been founded by a chronically infected individual[1]). Another recurrent mutation, nsp6:L37F, is associated with asymptomatic course of infection[20]; plausibly, it could have contributed to the ultimate recovery of the two immunocompromised patients in whom it has been observed (patient S and patient 3 from Truong et al., 2021).

Two of the mutations that emerged in patient S also spread in the general population as part of variants of concern (VOCs). The first is S:Δ69-70HV, which is a lineage-defining mutation of B.1.1.7. The second is S:L452R, which is found in several VOCs, including AY.1, AY.2 and B.1.617.2, as well as in multiple variants of interest. S:L452R was shown to have a pleiotropic effect, causing an escape both from T cell immunity and from neutralizing antibodies[11,21]. Finally, ORF8:Q18*, a stop-inducing mutation in ORF8, is reminiscent of the stop-inducing mutation in a different codon of the ORF8 protein (27th, as opposed to the 18th in this study) which is another of the lineage-defining mutations of B.1.1.7. The functions of ORF8 and its role in immune response and disease progression are extensively debated[20–22].

Supplementary Fig. 3. Patient S is robustly placed outside the cluster 5 clade. The abridged phylogeny of the B.1.1 lineage phylogeny is shown. Only those samples are shown which met either of the following conditions: (i) carried any of the differences found between the B.1.1 root and the patient S. sample (black cells), and these mutations had occurred in the branch immediately descendant from the B.1.1 root; or (ii) carried either the S:Δ69-70HV (blue cells) or the S:Y435F (yellow cells) mutation, independent of the timing of their origin. Additionally, we retained the samples from the branches that separate the cluster 5 clade from the rest of the phylogeny (two additional mutations, purple cells). Samples that didn't meet these criteria were collapsed, with the number of such samples shown in parentheses. The retained samples were then grouped by country, with names formatted as 'country|date of the earliest sample|number of samples'. B.1.1.7 and cluster 5 samples are shaded as in Fig. 1b. The presence of the above-mentioned mutations is indicated by the matrix at the right. Two mutations distinguishing cluster 5 from the B.1.1 root (purple) reject uniting patient S and cluster 5 in the same clade. For patient S, mutations with allele frequency below 50% in all three samples are shown in grey. Missing

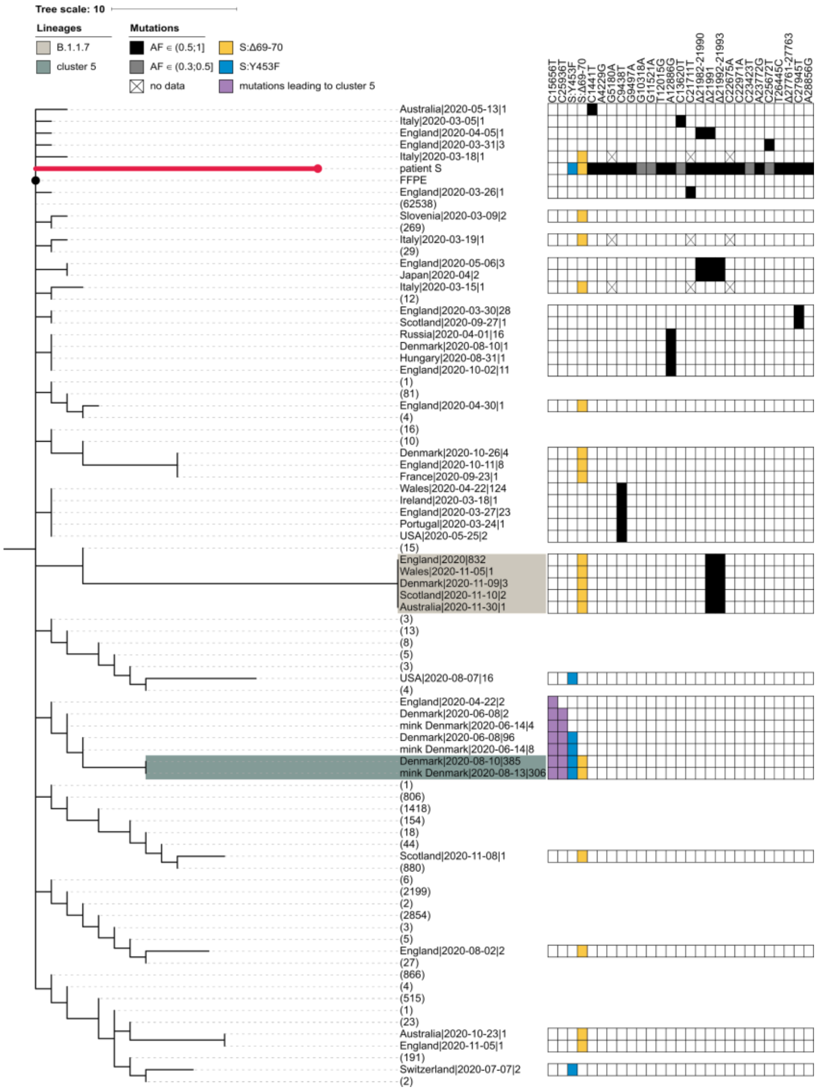

data (‘N’s in sequences) are shown as crosses. FFPE (black dot), patient A sample (the presumed source of infection for patient S).

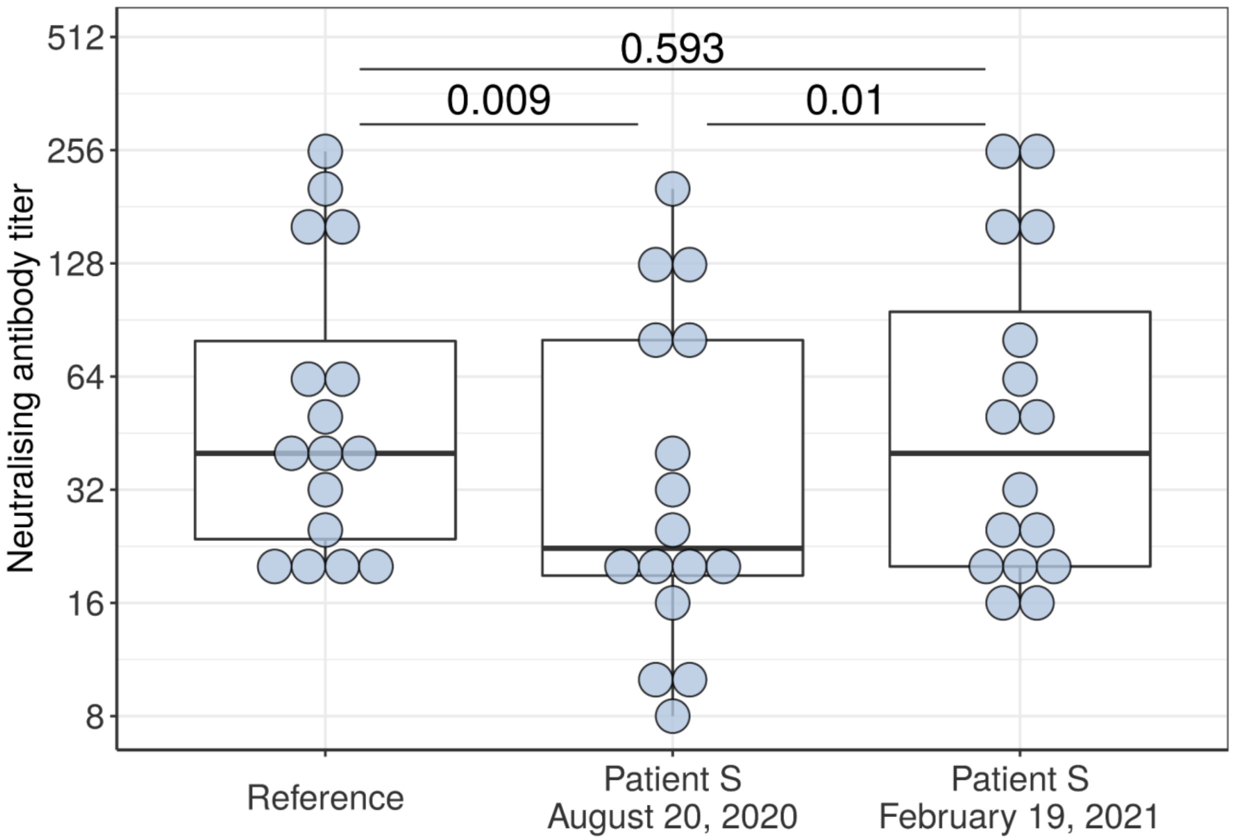

Supplementary Fig. 4 Effect of viral evolution in patient S on neutralization by antibodies. Neutralizing activity of serum obtained from 16 convalescent donors against patient S virus samples obtained on August 20, 2020 and February 19, 2021, as well as a reference viral strain of the B.1 lineage isolated from a swab sample in the beginning of the pandemic in Russia in March 2020. Each sample was tested in triplicate and GMTs are plotted. The pairwise comparison of the titers was performed via paired two-sided Mann-Whitney-Wilcoxon test with Holm multiple comparison adjustments. 95% confidence level was used. The August 20, 2020 isolate demonstrated significant reduction in sensitivity to neutralizing antibodies compared to the reference strain (location parameter 0.25, CI = [0.10-0.30], *W*-statistic = 112, $p = 0.003$, adjusted p = 0.009). Afterwards, by February 19 a sensitivity increased again (location parameter 0.20, CI = [-0.30, –0.10], *W*-statistic = 7, $p = 0.005$, adjusted $p = 0.01$). Consequently, February 19, 2021 isolate carried no signature of reduced sensitivity, and was indistinguishable in its sensitivity to neutralizing antibodies from the reference strain (location parameter $9.3x10^{-7}$, CI = [-0.10, 0.15], *W*-statistic = 39.5, $p = 0.593$, adjusted $p = 0.593$). Source data for are provided as a Source Data file.

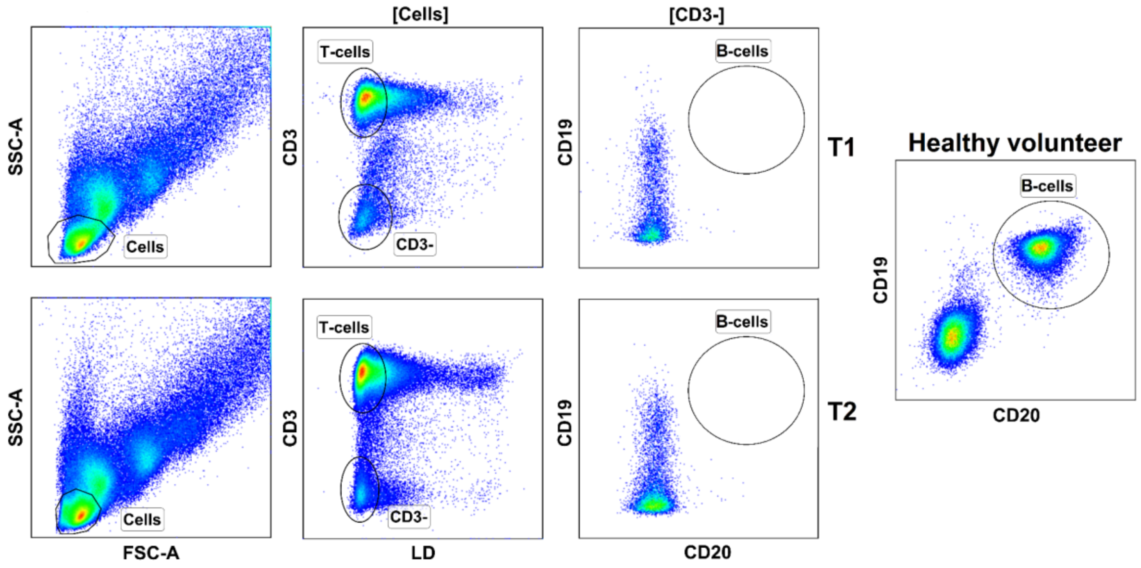

Supplementary Fig. 5 Flow cytometry plots showing the absence of B cells.

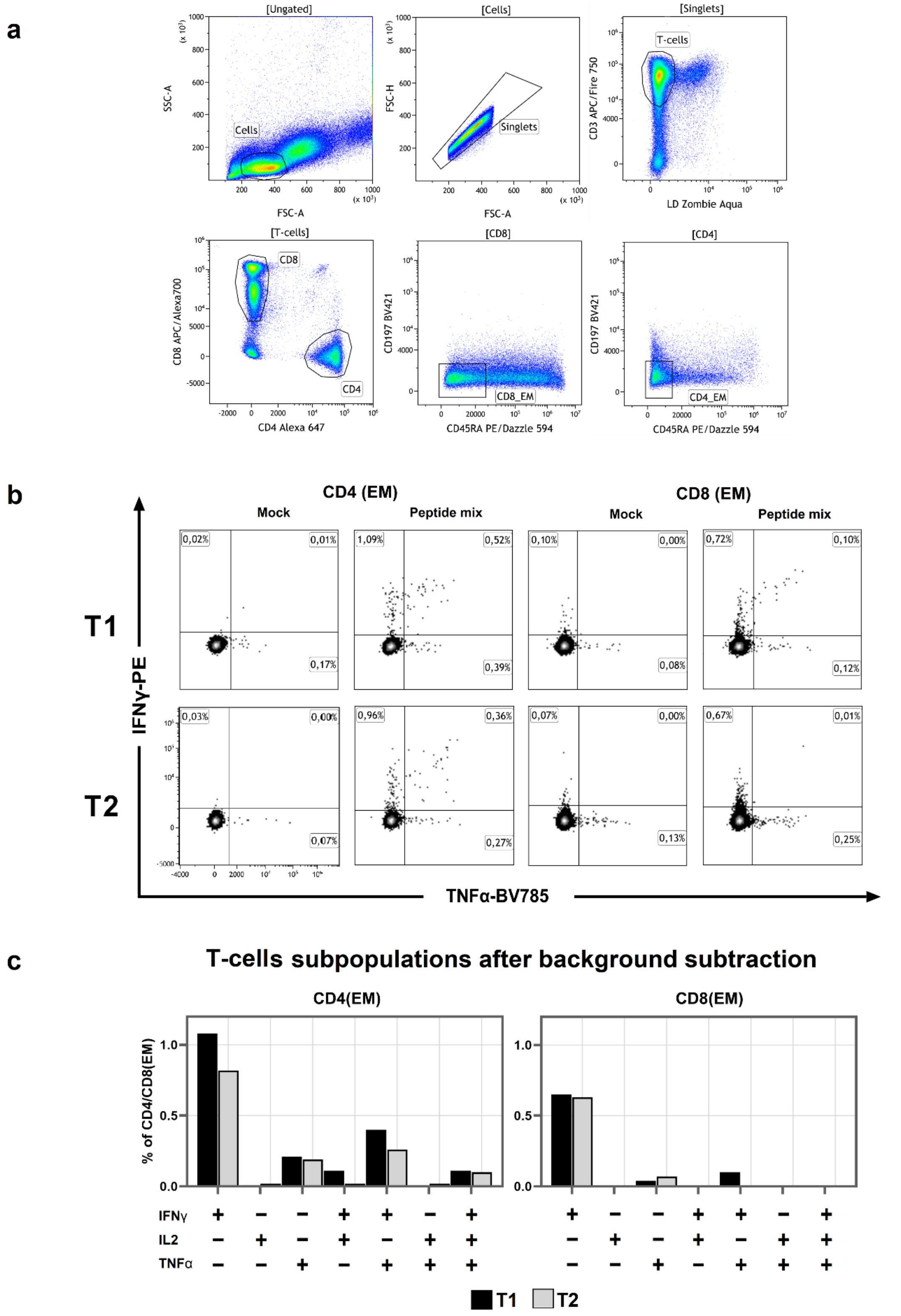
a
[Ungated]
SSC-A
FSC-A
Cells
[Cells]
FSC-H
Singlets
[Singlets]
CD3 APC/Fire 750
LD Zombie Aqua
T-cells
[T-cells]
CD8 APC/Alexa700
CD4 Alexa 647
CD8
CD4
[CD8]
CD197 BV421
CD45RA PE/Dazzle 594
CD8_EM
[CD4]
CD4_EM
b
CD4 (EM)
CD8 (EM)
Mock
Peptide mix
T1
T2
IFNγ-PE
TNFα-BV785
0,02%
0,01%
0,17%
1,09%
0,52%
0,39%
0,10%
0,00%
0,08%
0,72%
0,10%
0,12%
0,03%
0,00%
0,07%
0,96%
0,36%
0,27%
0,07%
0,00%
0,13%
0,67%
0,01%
0,25%
c
T-cells subpopulations after background subtraction
CD4(EM)
CD8(EM)
% of CD4/CD8(EM)
IFNγ
IL2
TNFα
T1
T2

Supplementary Fig. 6 Features of immune response in patient S. a: Gating strategy used to define CD4+ and CD8+ Tem cell populations, shown on the Figure 3 and the following panels of present Figure S6; **b:** CD4 and CD8 T-cell responses to peptide mixture of SARS-Cov-2 proteins S, N, M, ORF3a and ORF7a. Representative flow cytometry plots showing the cytokine profiles of SARS-CoV-2-specific CD4 and CD8 effector memory T cells after the stimulation; **c:** Bar-plots representing the percentage of different cytokine-producing populations of SARS-CoV-2-specific CD4 and CD8 T cells after background subtraction (data from the mock-stimulated sample were subtracted from peptide-stimulated samples). Time points T1 and T2 correspond to August 20, 2020 and February 16, 2021 respectively. Stimulation with peptide mixture of SARS-Cov-2 proteins S, N, M, ORF3a and ORF7a provoked expansion of both SARS-CoV-2-specific CD4 and CD8 T cells; the CD4 T-cell response predominated over CD8, as usual for COVID-19 patients [22,23].

Supplementary Table 3. **Results of HLA calling from WES data using HLA-HD.** Classical HLA genes A, B, C, DRB1 and DQB1 were additionally confirmed with HLA genotyping (see Methods). These alleles were further combined into haplotypes via HLA-2-Haplo software. Thus, haplotype 1 corresponds to A - 01:01, B - 08:01, C - 07:01, DRB1 - 03:01, DQB1 - 02:01 (population frequency 5.98e-2); and haplotype 2 corresponds to A - 03:01, B - 07:02, C - 07:02, DRB1 - 01:01, DQB1 - 05:01 (population frequency 3.37e-3). Worldwide allele frequencies are presented according to ref [24,25].

HLA	Allele 1	Frequency (Allele 1)	Allele 2	Frequency (Allele 2)
A	HLA-A*03:01:01	0.04272	HLA-A*01:01:01	0.04843
B	HLA-B*08:01:01	0.02960	HLA-B*07:02:01	0.04104
C	HLA-C*07:02:01	0.13101	HLA-C*07:01:01	0.06887
DRB1	HLA-DRB1*03:01:01	0.0676	HLA-DRB1*01:01:01	0.04123
DQA1	HLA-DQA1*01:01:01	-	HLA-DQA1*05:01:01	-
DQB1	HLA-DQB1*05:01:01	0.09307	HLA-DQB1*02:01:08	0.15003
DPA1	HLA-DPA1*01:03:01	-	-	-
DPB1	HLA-DPB1*04:02:01	0.18989	HLA-DPB1*04:01:01	0.23267

Supplementary Note 5

We excluded ORF8:Q18* from the analysis presented in Fig. 2 because it is impossible to calculate a presentation score (BR, PHBR or imBR) for a site lost from an amino acid sequence due to a stop gain. However, among the peptides absent from the ORF8 sequence after the ORF8:Q18* change, three were listed in IEDB as immunogenic on HLA I alleles carried by patient S, and one, as immunogenic on an HLA II allele carried by patient S. All of them are strong binders (Supplementary Table 4). Therefore, ORF8:Q18* also causes T cell escape.

Supplementary Table 4. **List of peptides experimentally validated in previous studies and included in the IEDB database that overlapped the mutations observed in this study.**

**Mutation**	**HLA class**	**Peptide before mutation**	**Peptide after mutation**	**HLA and change of percentile rank**
S:S50L	HLA I	FRSSVLH**S**T	FRSSVLH**L**T	HLA-C*07:01: Weak (0.74) -> Weak (0.93)
S:S50L	HLA I	**S**TQDLFLPF	**L**TQDLFLPF	HLA-A*01:01: Weak (1.1) -> Weak (1.6)
S:S50L	HLA I	**S**TQDLFLPFF	**L**TQDLFLPFF	HLA-A*01:01: Weak (1.2) -> Weak (1.7)
S:del140_144	HLA I	CNDP**FLGVY**	CNDPYHKNN	HLA-A*01:01: Strong (0.39) -> No binding
S:del140_144	HLA I	**GVY**YHKNNK	KNNKSWMES	HLA-A*03:01: Strong (0.046) -> No binding
S:del140_144	HLA I	FCNDP**FLGVY**Y	FCNDPYHKNNK	HLA-A*01:01: Weak (0.59) -> No binding
S:R273S	HLA I	YLQP**R**TFLL	YLQP**S**TFLL	HLA-B*08:01: Strong (0.019) -> Weak (0.69)
S:T470N	HLA I	KPFERDIS**T**EI	KPFERDIS**N**EI	HLA-B*07:02: Strong (0.11) -> Strong (0.15)
nsp3:T504A	HLA I	**T**DNYITTY	**A**DNYITTY	HLA-A*01:01: Strong (0.14) -> Weak (0.72)
nsp3:T504A	HLA I	P**T**DNYITTY	P**A**DNYITTY	HLA-A*01:01: Strong (0.007) -> Strong (0.07)
nsp3:T504P	HLA I	**T**DNYITTY	**P**DNYITTY	HLA-A*01:01: Strong (0.14) -> No binding
nsp3:T504P	HLA I	P**T**DNYITTY	P**P**DNYITTY	HLA-A*01:01: Strong (0.007) -> Weak (0.73)
nsp3:D821N	HLA I	TT**D**PSFLGRY	TT**N**PSFLGRY	HLA-A*01:01: Strong (0.001) -> Strong (0.068)
nsp3:D821N	HLA I	HTT**D**PSFLGRY	HTT**N**PSFLGRY	HLA-A*01:01: Strong (0.04) -> Strong (0.2)
nsp3:D821N	HLA I	TT**D**PSFLGRYM	TT**N**PSFLGRYM	HLA-A*01:01: Strong (0.089) -> Weak (1.2)

nsp3:T1456I	HLA I	S**T**NVTIATY	S**I**NVTIATY	HLA-A*01:01: Strong (0.097) -> Weak (0.67)
endornase:P205L	HLA I	K**P**RSQMEIDF	K**L**RSQMEIDF	HLA-B*07:02: Strong (0.3) -> No binding
ORF8:Q18*	HLA I	**QSCTQHQPY**	-	HLA-A*01:01: Strong (0.48) -> Lost
ORF8:Q18*	HLA I	**EPKLGSLVV**	-	HLA-B*07:02: Strong (0.49) -> Lost
ORF8:Q18*	HLA I	**VDDPCPIHFY**	-	HLA-A*01:01: Strong (0.16) -> Lost
N:P6T	HLA I	G**P**QNQRNAPRITF	G**T**QNQRNAPRITF	HLA-B*07:02: Strong (0.49) -> No binding
M:L129R	HLA I	VPLHGTI**L**	VPLHGTI**R**	HLA-B*07:02: Strong (0.29) -> No binding
ORF8:Q18*	HLA II	**PCPIHFYSKWYIRVG**	-	HLA-DRB1*01:01: Strong (0.54) -> Lost

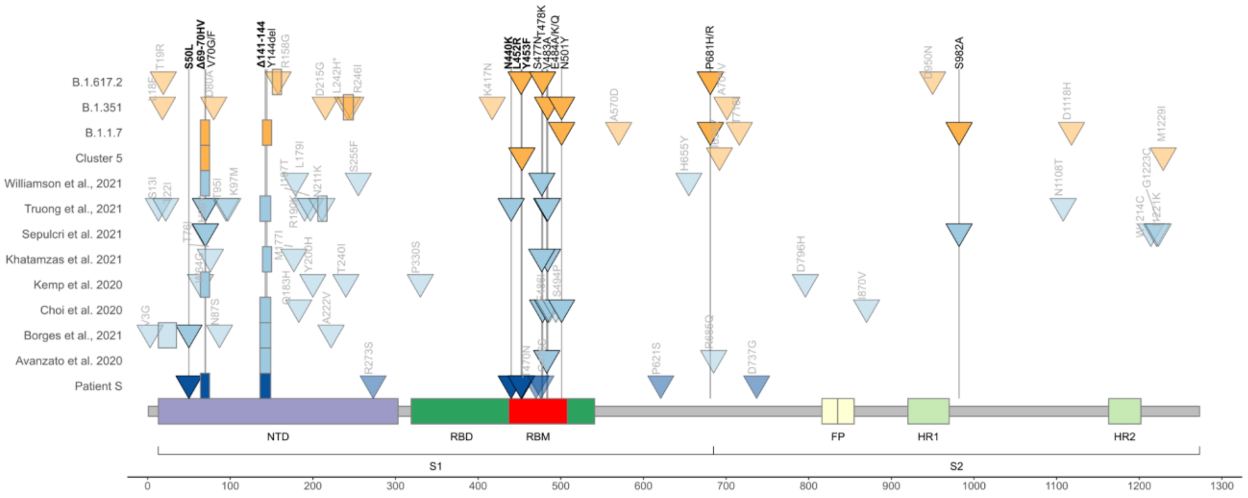

Supplementary Fig. 7. Concordant origin of spike mutations in notable COVID-19 variants and reported cases of persistent COVID-19. Shown are the locations of mutations in the amino acid sequence encoded by the spike gene. Rows, from top to bottom: VOCs Delta (B.1.617.2), Beta (B.1.351), Alpha (B.1.1.7); Cluster 5 variant; immunosuppressed individual with persistent infection for 290 days (Williamson et al., 2021); three patients with acute lymphoblastic leukemia who were persistently positive for SARS-CoV-2 (Truong et al., 2021); immunosuppressed individual treated with immunoglobulin (Sepulcri et al. 2021); immunosuppressed individual treated with convalescent plasma (Khatamzas et al. 2021); immunosuppressed individual treated with convalescent plasma (Kemp et al. 2020); immunosuppressed individual treated with Regeneron monoclonal antibody cocktail (Choi et al. 2020; only those mutations present at the final timepoint (T3, day 152) are shown); immunocompromised patient without convalescent plasma treatment (Borges et al., 2021); immunocompromised individual treated with convalescent plasma (Avanzato et al. 2020); immunosuppressed individual not treated with convalescent plasma or antibodies (patient S, this study). Triangles, point mutations; rectangles, deletions. Bright colors represent mutations observed in at least two studies. Mutations labeled on top in black were observed in multiple lineages/experiments, among those, mutations that are present in Patient S are highlighted with bold font.

Supplementary Reference

1. Cancer Therapy Adviser.
2. Simpson, S. *et al.* Radiological Society of North America Expert Consensus Document on Reporting Chest CT Findings Related to COVID-19: Endorsed by the Society of Thoracic Radiology, the American College of Radiology, and RSNA. *Radiol. Cardiothorac. Imaging* **2**, e200152 (2020).
3. Wu, F. *et al.* A new coronavirus associated with human respiratory disease in China. *Nature* **579**, 265–269 (2020).
4. Sergei Pond. Evolutionary annotation of global SARS-CoV-2/COVID-19 genomes enabled by data from GISAID. *observablehq.com* (2020).
5. Kosakovsky Pond, S. L. & Frost, S. D. W. Not So Different After All: A Comparison of Methods for Detecting Amino Acid Sites Under Selection. *Mol. Biol. Evol.* **22**, 1208–1222 (2005).
6. Oude Munnink, B. B. *et al.* Transmission of SARS-CoV-2 on mink farms between humans and mink and back to humans. *Science* **371**, 172–177 (2021).
7. Ria Lassaunière, Jannik Fona, Morten Rasmussen, Anders Frische, Charlotta Polacek Strandh, Thomas Bruun Rasmussen, Anette Bøtner, Anders Fomsgaard ger. Working paper on SARS-CoV-2 spike mutations arising in Danish mink, their spread to humans and neutralization data. (2020).
8. Chen, J., Wang, R., Wang, M. & Wei, G.-W. Mutations Strengthened SARS-CoV-2 Infectivity. *J. Mol. Biol.* **432**, 5212–5226 (2020).

9. Starr, T. N. *et al.* Prospective mapping of viral mutations that escape antibodies used to treat COVID-19. *Science* **371**, 850–854 (2021).
10. Betrains, A. *et al.* Convalescent plasma treatment of persistent severe acute respiratory syndrome coronavirus- 2 (SARS- CoV- 2) infection in patients with lymphoma with impaired humoral immunity and lack of neutralising antibodies. *Br. J. Haematol.* **192**, 1100–1105 (2021).
11. Truong, T. T. *et al.* Increased Viral Variants in Children and Young Adults with Impaired Humoral Immunity and Persistent Sars-Cov-2 Infection: A Consecutive Case Series. *EBioMedicine* **67**, 103355 (2021).
12. Kemp, S. *et al.* *Neutralising antibodies in Spike mediated SARS-CoV-2 adaptation*. http://medrxiv.org/lookup/doi/10.1101/2020.12.05.20241927 (2020) doi:10.1101/2020.12.05.20241927.
13. McCarthy, K. R. *et al.* Recurrent deletions in the SARS-CoV-2 spike glycoprotein drive antibody escape. *Science* **371**, 1139–1142 (2021).
14. Andrew Rambaut, Nick Loman, Oliver Pybus, Wendy Barclay, Jeff Barrett, Alesandro Carabelli, Tom Connor, Tom Peacock, David L Robertson, Erik Volz, on behalf of COVID-19 Genomics Consortium UK (CoG-UK). Preliminary genomic characterisation of an emergent SARS-CoV-2 lineage in the UK defined by a novel set of spike mutations. *Virological* (2020).
15. Khatamzas, E. *et al. Emergence of multiple SARS-CoV-2 mutations in an immunocompromised host*. http://medrxiv.org/lookup/doi/10.1101/2021.01.10.20248871 (2021) doi:10.1101/2021.01.10.20248871.
16. Agerer, B. *et al.* SARS-CoV-2 mutations in MHC-I-restricted epitopes evade CD8 $^{+}$ T cell responses. *Sci. Immunol.* **6**, eabg6461 (2021).
17. Dolton, G. *et al. Emergence of immune escape at dominant SARS-CoV-2 killer T-cell epitope*. http://medrxiv.org/lookup/doi/10.1101/2021.06.21.21259010 (2021) doi:10.1101/2021.06.21.21259010.
18. Wang, R., Chen, J., Hozumi, Y., Yin, C. & Wei, G.-W. Decoding Asymptomatic COVID-19 Infection and Transmission. *J. Phys. Chem. Lett.* **11**, 10007–10015 (2020).
19. Focosi, D. & Maggi, F. Neutralising antibody escape of SARS- CoV- 2 spike protein: Risk assessment for antibody- based Covid- 19 therapeutics and vaccines. *Rev. Med. Virol.* rmv.2231 (2021) doi:10.1002/rmv.2231.
20. Pereira, F. Evolutionary dynamics of the SARS-CoV-2 ORF8 accessory gene. *Infect. Genet. Evol.* **85**, 104525 (2020).
21. Zinzula, L. Lost in deletion: The enigmatic ORF8 protein of SARS-CoV-2. *Biochem. Biophys. Res. Commun.* **538**, 116–124 (2021).
22. Zhang, Y. *et al.* The ORF8 protein of SARS-CoV-2 mediates immune evasion through down-regulating MHC-I. *Proc. Natl. Acad. Sci.* **118**, e2024202118 (2021).
23. Habel, J. R. *et al. Suboptimal SARS-CoV-2-specific CD8* $^{+}$ *T-cell response associated with the prominent HLA-A*02:01 phenotype*. http://medrxiv.org/lookup/doi/10.1101/2020.08.17.20176370 (2020) doi:10.1101/2020.08.17.20176370.
24. Solberg, O. D. *et al.* Balancing selection and heterogeneity across the classical human leukocyte antigen loci: A meta-analytic review of 497 population studies. *Hum. Immunol.* **69**, 443–464 (2008).
25. Sarkizova, S. *et al.* A large peptidome dataset improves HLA class I epitope prediction across most of the human population. *Nat. Biotechnol.* **38**, 199–209 (2020).
