# Supplementary material for: SARS-CoV-2 escape from cytotoxic T cells during long-term COVID-19
Source: Nat Commun. 2023 Jan 10;14:149. doi: 10.1038/s41467-022-34033-x (PMC9831376; doi:10.1038/s41467-022-34033-x)
Supplement: Supplementary file 3 — Description of Additional Supplementary Files [file 41467_2022_34033_MOESM3_ESM.docx]

**Description of Additional Supplementary Files**

**File Name: Supplementary Data 1
Description:** SRA: Accession codes and sample description of raw sequencing data of SARS-CoV-2, obtained in the study and uploaded in Sequencing Read Archive; GISAID - patient S: Accession codes and sample description of consensus SARS-CoV-2 genome, obtained in the study and uploaded in GISAID; GISAID - All: Accession codes of SARS-CoV-2 genome sequences, used in the study from the GISAID database; Reagents: The list of reagents, used in the study; Epitopes ID: IEDB Identifiers of immunogenic T cell epitopes, analyzed in the study.
