# Supplementary material for: SARS-CoV-2 escape from cytotoxic T cells during long-term COVID-19
Source: Nat Commun. 2023 Jan 10;14:149. doi: 10.1038/s41467-022-34033-x (PMC9831376; doi:10.1038/s41467-022-34033-x)
Supplement: Supplementary file 5 — Reporting Summary [file 41467_2022_34033_MOESM5_ESM.pdf]

nature portfolio

Corresponding author(s): Evgeniia I. Alekseeva, Georgii A. Bazykin

Last updated by author(s): Oct 10, 2022

Reporting Summary

Nature Portfolio wishes to improve the reproducibility of the work that we publish. This form provides structure for consistency and transparency in reporting. For further information on Nature Portfolio policies, see our Editorial Policies and the Editorial Policy Checklist.

Statistics

For all statistical analyses, confirm that the following items are present in the figure legend, table legend, main text, or Methods section.

n/a	Confirmed	
☐	☒	The exact sample size (*n*) for each experimental group/condition, given as a discrete number and unit of measurement
☐	☒	A statement on whether measurements were taken from distinct samples or whether the same sample was measured repeatedly
☐	☒	The statistical test(s) used AND whether they are one- or two-sided<br>*Only common tests should be described solely by name; describe more complex techniques in the Methods section.*
☐	☒	A description of all covariates tested
☐	☒	A description of any assumptions or corrections, such as tests of normality and adjustment for multiple comparisons
☐	☒	A full description of the statistical parameters including central tendency (e.g. means) or other basic estimates (e.g. regression coefficient) AND variation (e.g. standard deviation) or associated estimates of uncertainty (e.g. confidence intervals)
☐	☒	For null hypothesis testing, the test statistic (e.g. *F*, *t*, *r*) with confidence intervals, effect sizes, degrees of freedom and *P* value noted<br>*Give P values as exact values whenever suitable.*
☒	☐	For Bayesian analysis, information on the choice of priors and Markov chain Monte Carlo settings
☒	☐	For hierarchical and complex designs, identification of the appropriate level for tests and full reporting of outcomes
☒	☐	Estimates of effect sizes (e.g. Cohen's *d*, Pearson's *r*), indicating how they were calculated

Our web collection on statistics for biologists contains articles on many of the points above.

Software and code

Policy information about availability of computer code

Data collection	CytExpert (Beckman Coulter)
Data analysis	Kaluza Analysis version 2.1; Trimmomatic version 0.39; BWA MEM version 0.7.17; ivar version 1.3; bcftools version 1.9; LoFreq version 2.1.5; MAFFT v7.453; Pangolin version 2.1.0; IQ-Tree v2.1.1; TreeTime v. 0.8.0; FastTree version 2.1.11; HLA-HD version 1.3.0; HLA-2-Haplo (web application, no versions https://hla.univ-nantes.fr/); netMHCpan version 4.1; netMHCIIpan version 4.0; R version 4.0.0; ggplot2 version 3.3.2; ITOL version 6. The code used in the study is available at https://github.com/EvgeniiaAlekseeva/patient_S, DOI: 10.5281/zenodo.77149702.

For manuscripts utilizing custom algorithms or software that are central to the research but not yet described in published literature, software must be made available to editors and reviewers. We strongly encourage code deposition in a community repository (e.g. GitHub). See the Nature Portfolio guidelines for submitting code & software for further information.

Data

Policy information about availability of data

All manuscripts must include a data availability statement. This statement should provide the following information, where applicable:

- Accession codes, unique identifiers, or web links for publicly available datasets
- A description of any restrictions on data availability
- For clinical datasets or third party data, please ensure that the statement adheres to our policy

Sequence data is available from the Sequence Read Archive: https://www.ncbi.nlm.nih.gov/bioproject/PRJNA749008/ (SRA: PRJNA749008, Supplementary Data 1). Consensus sequences are available from the GISAID with identifiers, presented in Supplementary Data 1. The list of peptides shown to be immunogenic to T cells in

other SARS-CoV-2 infected patients carrying the same HLA alleles as patient S, was downloaded from IEDB (Immune Epitope Database and Analysis Resource, accessed on June 1, 2021) with the "positive assay only" filter (https://www.iedb.org/). IEDB identifiers of found epitopes are presented in the Supplementary Data file.

Human research participants

Policy information about studies involving human research participants and Sex and Gender in Research.

Reporting on sex and gender	The sex was self-reported by the participant and was not relevant for the design or conclusions of the study.
Population characteristics	Patient S, a female in her 47 previously diagnosed with Non-Hodgkin's diffuse B-cell lymphoma IV stage B, tested positive for SARS-CoV-2 for the first time on April 17, 2020.
Recruitment	The work is based on the analysis, performed on a single patient. The patient had special immune system conditions and unique longevity of COVID-19, this is why the study was done on this specific patient. Thus, since the patients was involved in the study very specifically, there is no selection bias in this study.
Ethics oversight	Our research complies with the Local Ethics Review Board of the Smorodintsev Research Institute of Influenza (approved on April 30, 2020, reference number: 131) and by the Biomedical Ethics Committee of the I.P. Pavlov First Saint Petersburg State Medical University. The research protocol was approved by IRB and ethics committees and participants gave written informed consent, according to CARE guidelines (See: https://www.care-statement.org/), and in compliance with the Declaration of Helsinki principles.

Note that full information on the approval of the study protocol must also be provided in the manuscript.

Field-specific reporting

Please select the one below that is the best fit for your research. If you are not sure, read the appropriate sections before making your selection.

[x] Life sciences [ ] Behavioural & social sciences [ ] Ecological, evolutionary & environmental sciences

For a reference copy of the document with all sections, see nature.com/documents/nr-reporting-summary-flat.pdf

Life sciences study design

All studies must disclose on these points even when the disclosure is negative.

Sample size	This is a case study of long-term evolution of SARS-CoV-2 inside a single host with specific features of immune system (non-Hodgkin's lymphoma) and therapy design (rituximab + absence of convalescent plasma during most of the therapy). Such conditions together with the longevity of the disease (this is one of the longest cases described to date) make such cases very rare and hard to observe. Thus, the sample size of this study is limited by the single patient.
Data exclusions	Mutations detected in regions with poor read coverage (less than 4 reads) or with low read frequency less than 30% were excluded from the analyses.
Replication	We performed six whole-genome sequencing of SARS-CoV-2, obtained from nasopharyngeal swab samples from the patient in the period from August 17, 2020 to February 19, 2021. All mutations were detected in at least two of these samples with more than 30% read frequency (see Extended Data Table 2). All attempts at replication were successful and presented, included in data analysis and are available at GISAID.
Randomization	There were no experimental groups in this case study.
Blinding	Since this study investigates the rare case of long-term COVID-19 in a patient with specific immune conditions, it was impossible to form control and experimental groups of patients. Thus, blinding was not relevant in our case.

Reporting for specific materials, systems and methods

We require information from authors about some types of materials, experimental systems and methods used in many studies. Here, indicate whether each material, system or method listed is relevant to your study. If you are not sure if a list item applies to your research, read the appropriate section before selecting a response.

Materials & experimental systems

n/a	Involved in the study
[ ]	[x] Antibodies
[ ]	[x] Eukaryotic cell lines
[x]	[ ] Palaeontology and archaeology
[x]	[ ] Animals and other organisms
[x]	[ ] Clinical data
[x]	[ ] Dual use research of concern

Methods

n/a	Involved in the study
[x]	[ ] ChIP-seq
[ ]	[ ] Flow cytometry
[x]	[ ] MRI-based neuroimaging

Antibodies

Antibodies used

For B cells:
1. Zombie Aqua, Biolegend, Cat. # 423102, Lot. # B275859;
2. APC/Fire™ 750 anti-human CD19, Biolegend, Clone SJ25C1, Cat. # 363030, Lot. # B272420;
3. Brilliant Violet 421™ anti-human CD20, Biolegend, Clone 2H7, Cat. # 302330, Lot. # B253528;
4. Brilliant Violet 605™ anti-human CD3,Biolegend, Clone OKT3, Cat. # 317322, Lot. # B278819;

For T cells:

5. Zombie Aqua, Biolegend, Cat. # 423102, Lot. # B275859;
6. FITC anti-human IL-2, Biolegend, Clone MQ1-17H12, Cat. # 500304, Lot. # B268803;
7. PE anti-human IFN-, BC, Clone 45.15, Cat. # IM2717U, Lot. # 200046;
8. PE/Dazzle™ 594 anti-human CD45RA, Biolegend, Clone HI100, Cat. # 304146, Lot. # B294992;
9. Alexa Fluor® 647 anti-human CD4, Biolegend, Clone SK3, Cat. # 344636, Lot. # B304572;
10. Alexa Fluor® 700 anti-human CD8a, Biolegend, Clone HIT8a, Cat. # 300920, Lot. # B301545;
11. APC/Fire™ 750 anti-human CD3, Biolegend, Clone SK7, Cat. # 344840, Lot. # B286176;
12. Brilliant Violet 421™ anti-human CD197 (CCR7), BD, Clone 150503, Cat. # 562555, Lot. # 9304147;
13. Brilliant Violet 785™ anti-human TNF-, Biolegend, Clone MAb11, Cat. # 502948, Lot. # B275044;
14. Co-Stimulatory Antibodies (CD28/CD49d), BD, Clones L293/L25, Cat. #347690, Lot. # 1131991.

Validation

For B cells:
1. https://www.biolegend.com/en-us/products/zombie-aqua-fixable-viability-kit-8444
2. https://www.biolegend.com/en-us/products/apc-fire-750-anti-human-cd19-antibody-13037
3. https://www.biolegend.com/en-us/products/brilliant-violet-421-anti-human-cd20-antibody-7192
4. https://www.biolegend.com/en-us/products/brilliant-violet-605-anti-human-cd3-antibody-7666

For T cells:

5. https://www.biolegend.com/en-us/products/zombie-aqua-fixable-viability-kit-8444
6. https://www.biolegend.com/en-us/products/fitc-anti-human-il-2-antibody-1349
7. https://www.mybeckman.ru/reagents/coulter-flow-cytometry/antibodies-and-kits/single-color-antibodies/ifn-gamma/im2717u
8. https://www.biolegend.com/en-us/products/pe-dazzle-594-anti-human-cd45ra-antibody-10236
9. https://www.biolegend.com/en-us/products/alexa-fluor-647-anti-human-cd4-antibody-12070
10. https://www.biolegend.com/en-us/products/alexa-fluor-700-anti-human-cd8a-antibody-3434
11. https://www.biolegend.com/en-us/products/apc-fire-750-anti-human-cd3-antibody-13004
12. https://www.bdbiosciences.com/en-us/products/reagents/flow-cytometry-reagents/research-reagents/single-color-antibodies-ruo/bv421-mouse-anti-human-cd197-ccr7.562555
13. https://www.biolegend.com/en-us/products/brilliant-violet-785-anti-human-tnf-alpha-antibody-12027
14. https://www.bdbiosciences.com/en-eu/products/reagents/flow-cytometry-reagents/clinical-discovery-research/multicolor-cocktails-and-kits-ruo-gmp/co-stimulatory-antibodies-cd28-cd49d.347690.

Eukaryotic cell lines

Policy information about cell lines and Sex and Gender in Research

Cell line source(s)	Vero cells were derived from ATCC (#CCL-81); Vero E6 cells were derived from IZSLER/IBVR (# BS CL 87).
Authentication	None of the cell lines used were authenticated.
Mycoplasma contamination	We confirm that all cell lines were tested negative for mycoplasma contamination.
Commonly misidentified lines (See ICLAC register)	No

Flow Cytometry

Plots

Confirm that:

- [x] The axis labels state the marker and fluorochrome used (e.g. CD4-FITC).
- [x] The axis scales are clearly visible. Include numbers along axes only for bottom left plot of group (a 'group' is an analysis of identical markers).
- [x] All plots are contour plots with outliers or pseudocolor plots.
- [x] A numerical value for number of cells or percentage (with statistics) is provided.

Methodology

Sample preparation	Flow cytometry assays were performed using cryopreserved PBMCs. Cells were isolated from patients' heparinized blood by gradient centrifugation with lymphocyte separation medium Lymphosep (BioWest), frozen in freezing medium containing 10% DMSO (AppliChem) in FBS (Gibco) and stored in liquid nitrogen until usage.
Instrument	CytoFlex flow cytometer (Beckman Coulter)
Software	CytExpert (Beckman Coulter) – data collection, Kaluza 2.1(Beckman Coulter) – data analysis
Cell population abundance	In the present work, experiments on cell sorting were not carried out.
Gating strategy	Lymphocytes were gated based on their size and granularity; B-cells were identified as a live CD3-/CD19+/CD20+ population. To identify Tem, lymphocytes were gated based on their size and granularity. Live CD3+T cells were identified and subdivided into CD4+ and CD8+ T cells. These populations were further subdivided based on the expression of CD45RA and CD197 (CCR7). CD3+CD4+ or CD3+CD8+ lymphocytes with the CD45RA-/CCR7- phenotype were considered Tem cells. Cut-off values for the definition of cytokine-producing T cell responses stimulated with SARS-CoV-2 peptides were ≥5 events and a ≥2-fold difference in the magnitude of TNF+, IFNγ+ or IL-2+ Tem cells compared to the non-stimulated control.

- [x] Tick this box to confirm that a figure exemplifying the gating strategy is provided in the Supplementary Information.
